# Dandy-Walker Syndrome: Delayed Acute Presentation With Unusual Symptoms

**DOI:** 10.7759/cureus.50262

**Published:** 2023-12-10

**Authors:** Fakhar Hayat, Mohamed Ismail, Muhanned M Alqhtani, Talal Almayman, Noor Sardar, Abdullah Ismaeel, Mohammed AlJohani, Rayan S Alruwaili

**Affiliations:** 1 Neurosurgery, King Hamad University Hospital, Busaiteen, BHR; 2 Neurological Surgery, King Hamad University Hospital, Busaiteen, BHR; 3 Pediatric Neurology, King Hamad University Hospital, Busaiteen, BHR; 4 Internal Medicine, Divisional Headquarter (DHQ) Teaching Hospital, Dera Ismail Khan, PAK; 5 General Surgery, Hafar Al-Batin Central Hospital, Hafar Al-Batin, SAU

**Keywords:** external ventricular drain (evd), trans-ependymal cerebrospinal fluid permeation, extrapyramidal symptoms, pediatric neurology, neurological manifestations, dandy walker syndrome

## Abstract

Dandy-Walker syndrome (DWS) is a rare congenital brain malformation defined by the presence of an expanded posterior fossa, full or partial absence of the cerebellar vermis, and a cystic expansion of the fourth ventricle. We report an 18-month-old girl with DWS presenting with atypical clinical manifestations and unusual symptoms. She initially presented with persistent vomiting and abdominal pain for four days, not responding to antiemetic medication. In addition, she was found to have abnormal postural arching of the back, extension of the lower limbs, and neck extension. MRI and CT head suggested Dandy-Walker syndrome with hydrocephalus (the lateral ventricle, third ventricle, and fourth ventricle are all significantly dilated with evidence of trans-ependymal cerebrospinal fluid permeation, severe compression anterior displacement of the brain stem). The patient underwent urgent, lifesaving right sub-occipital craniotomy, evacuation, and decompression of the posterior fossa cyst and external ventricular drain (EVD) insertion along with left supra-tentorial EVD insertion. A series of brain magnetic imaging and CT brain post-procedure studies showed a significant reduction in the size of the ventricular system and mass effect on the brain stem.

## Introduction

Dandy-Walker syndrome (DWS) is a rare congenital brain malformation defined by the presence of an expanded posterior fossa, full or partial absence of the cerebellar vermis, and a cystic expansion of the fourth ventricle [1]. Around 1 in 25,000 births suffer from DWS, and females are commonly affected more than males, with a ratio of 3:1 [2,3]. The estimated mortality rate is between 12% to 50% [4]. The cause of DWS is not fully understood; most cases of DWS are sporadic, but some can be caused by chromosomal abnormalities, genetic disorders, or environmental factors such as congenital TORCH (toxoplasmosis, other {syphilis, varicella-zoster, parvovirus B19}, rubella, cytomegalovirus, and herpes) infections or fetal alcohol syndrome [5]. In addition, DWS has been linked to a deletion of a region of chromosome 3 called 3q24q25.1. This deletion includes the ZIC1 and ZIC4 genes, which are thought to play a role in DWS. In mouse models, deleting these genes causes DWS-like symptoms [5]. Magnetic resonance imaging (MRI) of the brain is the best investigation modality used to diagnose DWS malformation and its variants, which include complete or partial agenesis of the cerebellar vermis, cystic dilatation of the fourth ventricle, and enlarged posterior fossa [1]. Early detection can be possible by transabdominal and transvaginal ultrasonography by visualization of the posterior fossa during antenatal visits in the first trimester of pregnancy [6]. Clinically, DWS presents with a wide variety of intensity and symptoms. Common characteristics include hydrocephalus (fluid retention in the brain), intellectual disability, developmental delay, muscle stiffness, trouble coordinating movements, and issues with balance and walking. The face, heart, limbs, and urinary system are among the other possible related disorders. The symptoms and related problems of DWS are the main focus of treatment. Physical and occupational therapy to enhance motor skills and coordination, surgery to treat hydrocephalus, and medication to control seizures or other neurological symptoms are all possible forms of treatment.

## Case presentation

**Figure 1:**
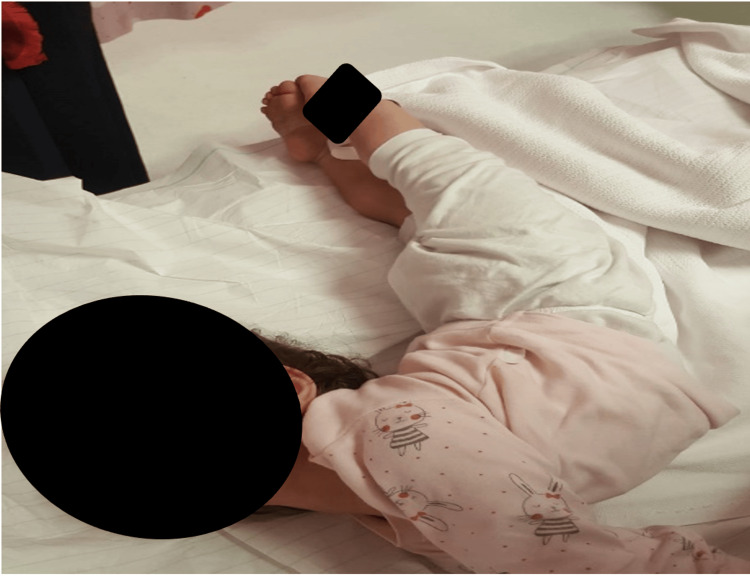
Abnormal postural arching of the back, extension of the lower limbs and neck.

An 18-month-old, previously healthy female child, previously healthy with normal motor development that corresponds to her age and no family history of neurological diseases was presented by the family to the emergency department with a four-day history of vomiting and abdominal pain associated with agitation and ataxia for two days. The patient was seen by the emergency physician, and supportive management was given. The patient was discharged on oral metoclopramide. Two days later, the child was brought back to the emergency department with complaints of persistent vomiting and progressively worsening ataxia, reduced activity, poor appetite, sleep disturbance, and restlessness. She gradually developed abnormal backward arching of the neck, trunk, and back, extension of the lower limbs, and hypertonia of all four limbs (Figure 1). She had generalized limb weakness and brisk reflexes. The child has been progressively unable to speak or interact with the family with a Glasgow Coma Scale (GCS) 8/15. Both pupils were equal and reactive to light and cranial nerves were intact. The impression was query encephalitis, acute dystonia, or drug-induced extrapyramidal symptoms.

**Figure 2:**
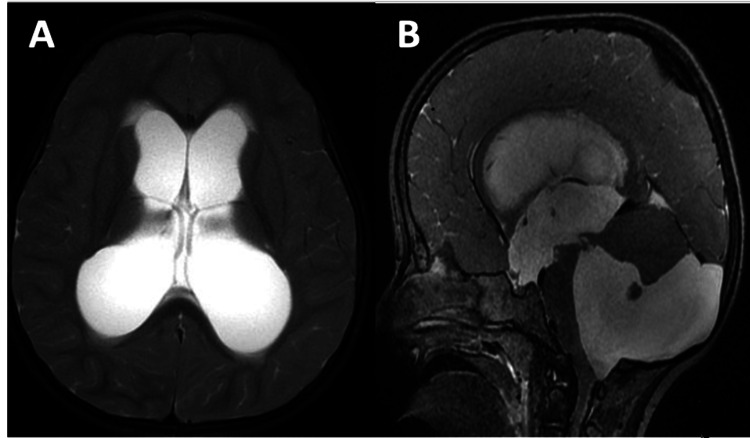
Preoperative MRI brain. (A) Axial cut image shows acute hydrocephalus with transependymal fluid permeation; (B) sagittal image shows large posterior fossa cyst communicating with the fourth ventricle severe compression of the brain stem, cerebellum, and vermis, as well as a dilated supratentorial ventricular system.

The patient was admitted to the pediatric intensive care unit (PICU) and she was intubated because of severe airway compromise by rigid extension posture and dystonia. The following drugs were given: intravenous diphenhydramine, ceftriaxone, acyclovir, midazolam, benzodiazepine, and loading and maintenance doses of levetiracetam. Urgent MRI and CT head showed significant dilatation of the lateral ventricles, third ventricle, and fourth ventricle, with evidence of trans-ependymal CSF permeation and periventricular brain edema and anterior displacement and compression of the brainstem, suggestive of DWS with acute hydrocephalus (Figure 2). There was no family history of neurological diseases, and developmental factors were age-appropriate.

**Figure 3:**
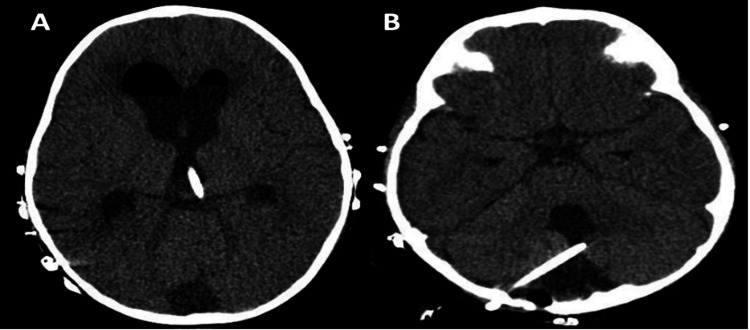
Postoperative CT brain scans. (A) Axial cut with marked reduced hydrocephalus changes and shunt in the lateral third ventricle; (B) axial cut with reduced posterior fossa size and the drain tip.

The patient clinical picture deteriorated when she was shifted to the operating theatre, her pupils were unequal, with the right pupil dilated to 5 mm and the left pupil dilated to 3 mm. The patient underwent urgent, lifesaving right sub-occipital craniotomy, evacuation, and decompression of the posterior fossa cyst and external ventricular drain (EVD) insertion along with left supra-tentorial EVD insertion. Intra-operative findings were consistent with extremely high intra-cystic pressure and related supratentorial and infratentorial intracranial pressure (ICP). Postoperatively, she could move all four limbs with tactile stimulation. She was kept on prophylactic anticonvulsants and antibiotics. She was kept in the critical care unit and gradually weaned off ventilator support. Her CSF analysis and cultures were within normal limits. Postoperative CT head showed a significant reduction in the size of the ventricular system, a mass effect on the brainstem, and improved brain edema (Figure 3). The child had generalized limbs with severe weakness of 2 to 3/5 Medical Research Council (MRC) power grading with coarse tremors. Her consciousness level gradually improved, and she could easily follow objects or light. Her pupils were both 2 mm and sluggishly reactive. She suffered focal motor seizures, developed exaggerated ankle clonus (++), and sustained clonus bilaterally in the lower limbs, which was managed with phenobarbital and levetiracetam. The ophthalmologic evaluation showed no signs of papilledema.

**Figure 4:**
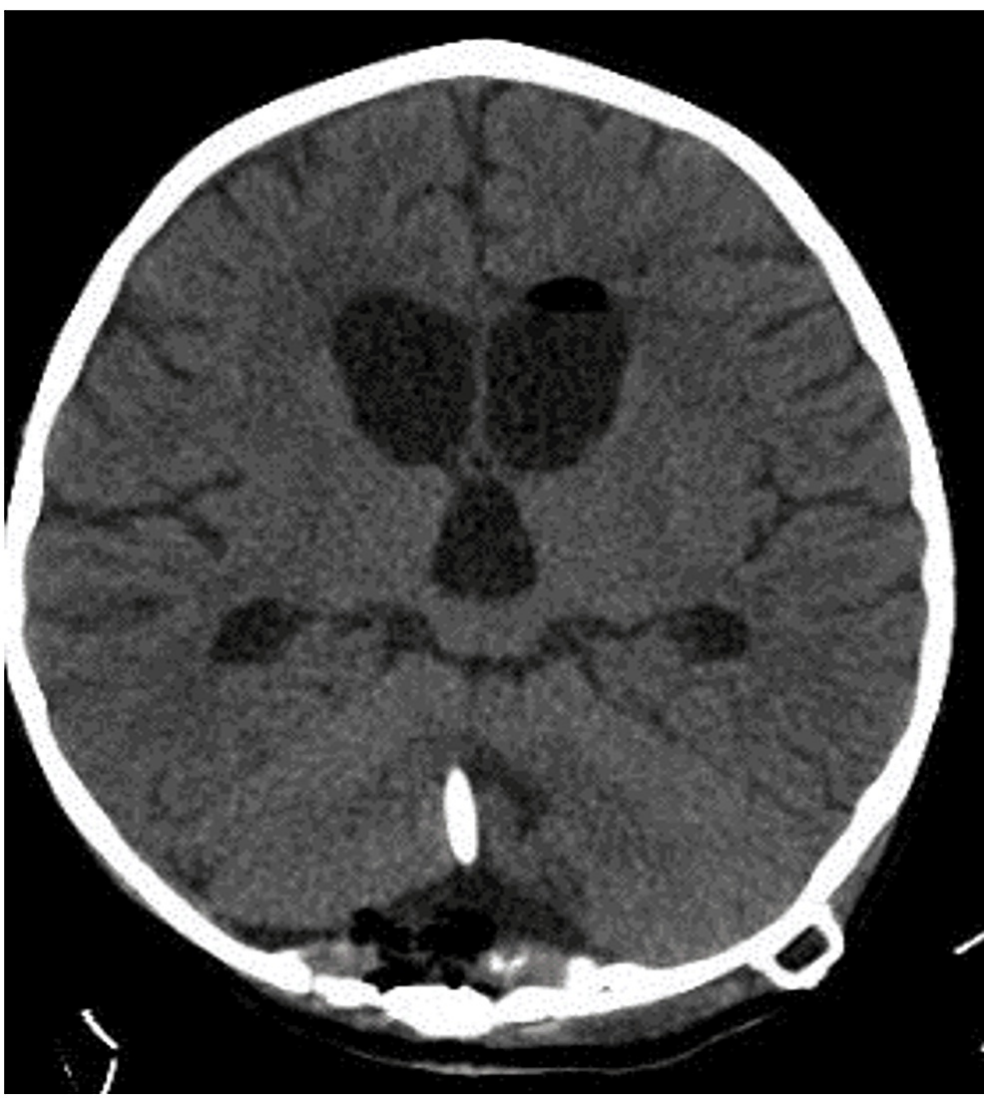
Postoperative CT brain scans. Axial cut with definitive ventriculoperitoneal shun tip and reservoir.

Supratentorial EVD was gradually closed and the follow-up serial CT head showed a decrease in the size of the temporal horns of the lateral ventricles and radiological resolution of features of supratentorial hydrocephalus (Figure 4). Therefore, a single posterior fossa CSF diversion shunt was planned. The patient underwent a suboccipital redo craniotomy, microscopic posterior fossa cyst fenestration, insertion of a posterior fossa ventriculoperitoneal (VP) shunt, and continued on anticonvulsants. After the operation, the patient was noted to have prolonged poor responsiveness and conscious level GCS 9-10/15, generalized weakness, and hypotonia. The patient's right lower limb and ankle were hypertonic, with exaggerated ankle reflex clonus (+). She had no facial expressions or oral movements.

Two weeks later, the patient's consciousness level improved, and she spontaneously opened her eyes, a good response to interaction with her parents, GCS 13/15. No abnormal movements or seizures were seen. Her tone and pupils were normal, but she still had exaggerated ankle reflexes. Postoperative CT brain scans showed a complete resolution of both supratentorial and infratentorial hydrocephalus. An EEG showed a recording of occipital region polymorphic, continuous, symmetrical, and reactive rhythm containing up to 3 Hz activity of moderate amplitude with normal changes in sleep. No epileptiform discharges were seen.

## Discussion

This case presents an unusual presentation of DWS, which is a rare congenital brain anomaly. Where 85% of cases are discovered before 1 year of age [1]. A routine prenatal sonography, done by obstetricians can usually detect this malformation during the later prenatal period [6]. With an incidence of approximately 1 in 30,000 cases, the clinical manifestations of hydrocephalus in DWS are diagnosed prior to the age of one year in 80% of patients [7]. The clinical presentation is often nonspecific, subject to multiple factors, including the severity of hydrocephalus and intracranial hypertension, and associated with comorbidities.

Variable symptoms like macrocephaly, delayed developmental milestones, irritability, vomiting, and convulsions may lead to initial presentation [8,9]. The most common manifestation is macrocephaly, which affects 90-100% of patients during their first months of life [10]. However, in this case, the patient did not present with any of these classic features until she was 18 months old. Another unusual aspect of this case is the patient's acute dystonic presentation. Dystonia is defined as “a movement disorder characterized by sustained or intermittent muscle contractions causing abnormal, often repetitive, movements, postures, or both” [11]. The patient's unusual presentation likely contributed to the delay in her diagnosis. Her initial symptoms of vomiting and abdominal pain were nonspecific and could have been caused by a variety of other conditions. Additionally, her dystonia was initially thought to be drug-induced. However, opisthosomas, extensor arching with the posturing of her head and spine is highly likely from meningeal irritation of the craniospinal junction from extremely high posterior fossa intracranial pressure and transmitted high intraspinal pressure.

Despite the delay in diagnosis, the patient received prompt and appropriate treatment. She underwent an urgent, life-saving suboccipital posterior fossa decompressive craniectomy and the insertion of right and left extra ventricular drains to relieve the hydrocephalus and cerebrospinal fluid diversion.

The endoscopic third ventriculostomy (ETV) became a well-established surgical procedure for treating obstructive hydrocephalus [12]. During the surgical procedure, an endoscope inserted via a burr hole into the ventricular system is used to create an aperture in the third ventricle's floor [12]. This bypasses any obstruction by enabling the cerebrospinal fluid to go straight to the basal cisterns. Aqueductal stenosis is one type of obstructive hydrocephalus that is used to be treated by ETV [12]. The patient also received anticonvulsants to control her seizures. As a result of her treatment, the patient's condition improved significantly and her Cognition improved to near normal, she regained complete motor power in all four limbs in the next 2 months with neuro-rehabilitation, cognitive and occupational therapy. 

This case highlights the importance of considering rare diagnoses, such as DWS, even in patients with atypical presentations. Additionally, it is important to be aware that dystonia can be a rare but serious complication of DWS in children.

## Conclusions

Dandy-Walker syndrome (DWS) is a rare neurodevelopmental disorder characterized by a large posterior fossa cyst, cerebellar vermis hypoplasia or agenesis, and fourth ventricle enlargement. Early recognition, comprehensive diagnostic evaluation, and multidisciplinary management are key to optimizing outcomes for individuals with DWS. Long-term follow-up is important to address developmental delays and associated medical conditions and provide appropriate support and interventions.
